# Recursive model for dose-time responses in pharmacological studies

**DOI:** 10.1186/s12859-019-2831-4

**Published:** 2019-06-20

**Authors:** Saugato Rahman Dhruba, Aminur Rahman, Raziur Rahman, Souparno Ghosh, Ranadip Pal

**Affiliations:** 1 Department of Electrical and Computer Engineering, Texas Tech University, 1012 Boston Ave, Lubbock, 79409 TX USA; 2 Department of Mathematics and Statistics, Texas Tech University, 1108 Memorial Circle, Lubbock, 79409 TX USA

**Keywords:** Drug sensitivity prediction, Pharmacogenomic studies, HMS-LINCS, Joint dose-time modeling, Recursive modeling, Dose-response curve, Tumor growth model, Gompertz law

## Abstract

**Background:**

Clinical studies often track dose-response curves of subjects over time. One can easily model the dose-response curve at each time point with Hill equation, but such a model fails to capture the temporal evolution of the curves. On the other hand, one can use Gompertz equation to model the temporal behaviors at each dose without capturing the evolution of time curves across dosage.

**Results:**

In this article, we propose a parametric model for dose-time responses that follows Gompertz law in time and Hill equation across dose approximately. We derive a recursion relation for dose-response curves over time capturing the temporal evolution and then specify a regression model connecting the parameters controlling the dose-time responses with individual level proteomic data. The resultant joint model allows us to predict the dose-response curves over time for new individuals.

**Conclusion:**

We have compared the efficacy of our proposed Recursive Hybrid model with individual dose-response predictive models at desired time points. We note that our proposed model exhibits a superior performance compared to the individual ones for both synthetic data and actual pharmacological data. For the desired dose-time varying genetic characterization and drug response values, we have used the HMS-LINCS database and demonstrated the effectiveness of our model for all available anticancer compounds.

**Electronic supplementary material:**

The online version of this article (10.1186/s12859-019-2831-4) contains supplementary material, which is available to authorized users.

## Background

One of the most important goal of precision medicine is to predict sensitivity of an anticancer drug to a given patient. Although, patients are most often characterized by their gene expressions, a more precise characterization is obtained by studying their proteomic expressions [1]. Harvard Medical School Library of Integrated Network-Based Cellular Signatures (HMS-LINCS) [2] offers proteomic data for cancer cell lines measured at various time intervals post drug application along with observed apoptosis fractions over different drug concentration and time. Given the evolution of protein expression over drug concentration and time, we would like to predict the apoptosis fractions of individuals over the same drug concentration at 72 hours post application of drug as 72 hours is considered to be the steady-state for cell viability studies [3, 4].

Our data, therefore, consists of a collection of temporally varying dose-response curves for each individual and the predictors are also a collection of temporally varying expressions, for multiple proteins, observed at different drug concentrations. This may appear as a standard function-on-function concurrent regression [5], but several obstacles arise, such as– (a) number of functional predictors exceeds the number of cell lines, (b) protein expression curves are observed at different time points as compared to the dose-response curves with only little overlap. Consequently, standard parametric statistical models cannot be readily applied here. Turning to model-free procedures, Matlock et al. [6] used Random Forest (RF) methodology to analyze the same HMS-LINCS data. While their methodology alleviates the *small sample-high dimension* problem, it cannot make temporal prediction of dose-response curves in absence of predictor information at the predicting time point. More precisely, given the observed dose-response curve of an individual at 48 hours, it cannot predict the curve at 72 hours. Therefore, the RF model either needs to wait till 72 hours and observe the protein expression curves to predict response, or it requires extrapolating protein expression data to 72 hours and then make predictions.

Standard machine learning (ML) approaches also fail to explicitly take into account a few well established properties of dose-response curves and their temporal evolution. For instance, *Haber’s law* suggest a monotonic relationship between responses observed at two successive time points at a given dose [7] and this rule, in turn, induces the dose-response curve observed at a later time point to dominate or be dominated by the curves observed at earlier time points [8]. Such constraints cannot be easily built into ML algorithms. Furthermore, these models provide little insight into the steady-state properties of the dose-response curves. Regardless of these shortcomings, several studies have demonstrated superior predictive performance of RF based models in drug sensitivity predictions [9–11].

To alleviate some theoretical restrictions of the foregoing ML approaches, while borrowing the predictive strength of RF methodology, we offer a hybrid model that satisfies some physical laws that dose-response curves are expected to satisfy while retaining a flexible model-free relationship between predictors and responses. In particular, we propose a parametric model for dose-time responses that follows the Gompertz law in time and approximately follows the Hill equation across dose. We derive a recursion relation for dose-response curves over time capturing the temporal evolution and theoretically examine their steady-state behavior. We then specify an RF model connecting the parameters controlling the dose-time responses with individual level proteomic data. The resultant joint model allows us to predict dose-response curves over time for new individuals. The complete fitting code along with a synthetic example can be obtained from GitHub via: https://github.com/dhruba018/Dose_time_Response_Recursive_Model.

## Results

We have evaluated the performance of our proposed recursive hybrid model using both synthetic data and HMS-LINCS database mentioned above. Note that, we were forced to limit our analysis to a single dataset since, to our knowledge, HMS-LINCS is the only publicly available source offering functional responses as well as predictors. Furthermore, dimensions of HMS-LINCS datasets are restricted to a handful of drugs and samples with a higher number of predictors in contrast to some common pharmacogenomics databases (*e.g.*, CCLE [12] or GDSC [13]) that provides dose-response curves for hundreds of samples, but with static feature sets. Here, we use HMS-LINCS as our synthetic data generation baseline first and then directly for analysis.

### Description of HMS-LINCS datasets

We used two distinct datasets from HMS-LINCS as our predictor and response sets. The predictor set consists of dose-time expression for 21 proteins and the response set contains the mean apoptosis fractions observed in 10 BRAF ^V600E/D^ melanoma cell lines over multiple doses and time points [2, 6]. Both protein expressions and apoptosis fractions are available post drug application at 7 dose levels ranging from 3.2 *nM* to 3.2 *μM*. However, while the protein expressions are available at 5 different time points (1,5,10,24 & 48 hours post drug application), the apoptosis data is available only for 24,48 & 72 hours post drug application. Apoptosis fraction was computed from the number of apoptotic cells at each dose-time point and the total initial number of cells normalized with DMSO control and then averaged over 4 replicates to produce the mean value. Both sets are available for 5 RAF/MEK inhibitor drugs. More detailed descriptions can be obtained from [2, 6].

For this study, we only use protein expressions observed at 24 & 48 hours, since those expressions match the temporal record of the responses (24,48 & 72 hours). For the 72 hours scenario, we use a time series model for data extrapolation (elaborate description is provided below in “Application on HMS-LINCS data” section). Moreover, only 14 out of 21 proteins have complete records in the covariate set, therefore, we only use these 14 proteins as our final predictors.

### Simulation study

To demonstrate the efficacy of our proposed model, we have performed a simulation study involving two synthetic datasets with 7 subjects, 7 dose levels, and 8 time points each (*m*=7, *D*=7, *T*=8). The predictor set contains the expression of 14 different covariates at each dose-time point (*P*=14). For detailed explanation of the terms used here, look into the “Methods” section.

#### Synthetic data generation

We first form the slope coefficient matrix in Eq. (20) by using the difference between HMS-LINCS protein expressions $x_{t, d, i}^{(p)}$ at 24 & 48 hours post application of the drug AZ-628 as the base ***β***_*d*,*i*_ and add random noise to create distinction between subjects. This yields a (***β***_*d*,*i*_)_49 × 14_ matrix per subject (for 7 time point differences and 7 doses). We also use the 24 hour expression data post AZ-628 application in cell line C32 as baseline covariates and add random noise to create our predictors per subject $z_{t, d, i}^{(p)}, \quad p = 1, 2, \cdots, 14$.

1 $$\begin{array}{*{20}l}  {} \beta_{d, i}^{(p)} &\!:=\! \frac{dx_{\cdot, d, i}^{(p)}}{dt} \,=\, x_{48, d, i}^{(p)} \,-\, x_{24, d, i}^{(p)} + \nu_{i}, \ \ \nu_{i} \sim \mathcal{U} \big(-0.01, 0.02 \big) \end{array} $$

2 $$\begin{array}{*{20}l} {}z_{0, d, i}^{(p)} &= x_{24, d, C32}^{(p)} + \nu'_{i}, \qquad \nu'_{i} \sim \mathcal{U} \big(-0.5, 0.5 \big) \\ {}z_{t, d, i}^{(p)} &= z_{t^{-}, d, i}^{(p)} + \beta_{d, i}^{(p)} \Big\vert_{\Delta t \, = \, t \, - \, t^{-}}, \qquad t = 1, 2, \cdots, 7 \end{array} $$

**Fig. 1:**
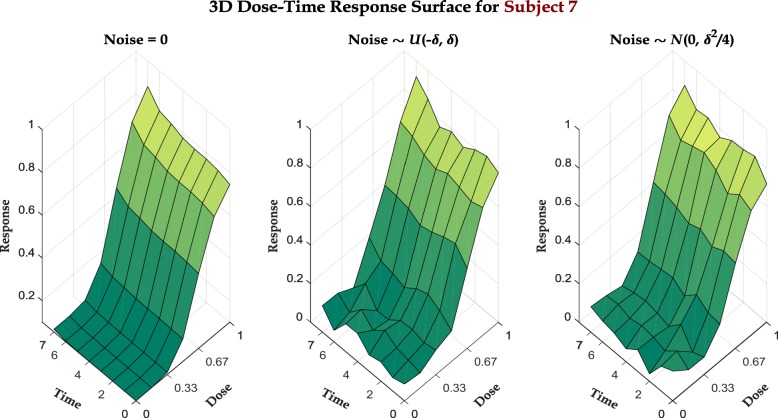
Dose-time response surfaces for synthetic data under various additive noise conditions

For the output, we create a response matrix ***V***_8 × 7_ (*D*=7, *T*=8) per subject where dose-response values for the initial time epoch are extracted from the 4 parameter sigmoidal dose-response model *g*(*d*) in Eq. (8) assuming *a*, *b* and *θ* as fixed but *c*_*i*_ (*i.e.*, *EC*_50_) to be varying with each subject *i*. To generate the responses for the remaining 7 time points, we estimate the growth and scaling coefficients (*α*_*d*,*i*_, *γ*_*d*,*i*_ in (20)) as linear models of the slope coefficient vector ***β***_*d*,*i*_ with random weight vector sets and take the final estimates as the maxima of the estimates at current and immediately previous doses (following property (ii) of the recursive model in (14)). We assume the 7 dose levels to be linearly spread in the interval [0,1].

3 $$ {}\begin{aligned} {\alpha}_{d, i} &\!= w_{\alpha, i}^{(0)} + w_{\alpha, i}^{(1)} \beta_{d, i}^{(1)} + \cdots + w_{\alpha, i}^{(14)} \beta_{d, i}^{(14)} = w_{\alpha, i}^{(0)} + \boldsymbol{\beta}_{d, i} \boldsymbol{w}_{\alpha, i} \\ {\gamma}_{d, i} &\!= w_{\gamma, i}^{(0)} + w_{\gamma, i}^{(1)} \beta_{d, i}^{(1)} + \cdots + w_{\gamma, i}^{(14)} \beta_{d, i}^{(14)} = w_{\gamma, i}^{(0)} + \boldsymbol{\beta}_{d, i} \boldsymbol{w}_{\gamma, i} \\ \hat{\alpha}_{d, i} &\,=\, \max\!\big(\!\alpha_{d, i},\!\alpha_{d^{-}, i\!} \big), \ \ \hat{\gamma}_{d, i} = \max\!\big(\gamma_{d, i},\!\gamma_{d^{-}, i} \big), \ \ d = 2, \cdots, 7 \\ \end{aligned}  $$

where $w_{\cdot, i}^{(p)} \sim \mathcal {U}(0, 1)$ are randomly chosen weight values for protein *p* in subject *i*. We then use this $\hat {\alpha }_{d, i},\ \hat {\gamma }_{d, i}$ in the one-step prediction relation in Eq. (21) to generate responses at *t*>0. Figure 1 illustrates the monotonicity of drug response surfaces over dose and time for a representative subject with different levels of additive noise in response. An additional section displays the dose-time response surfaces for all 7 subjects [see Additional file 1: Figures S1-S3].

4 $$  \begin{aligned} \tilde{\alpha}_{d, i} &= \hat{\alpha}_{d, i} \Big\vert_{\Delta t \, = \, t \, - \, t^{-}} \quad \tilde{\gamma}_{d, i} = \hat{\gamma}_{d, i} \Big\vert_{\Delta t \, = \, t \, - \, t^{-}} \\ v_{t, d, i} &= v_{t^{-}, d, i} \; e^{\, \tilde{\gamma}_{d, i} \left(1 \, - \, e^{-\tilde{\alpha}_{d, i}} \right)}, \quad t = 1, 2, \cdots, 7 \end{aligned}  $$

#### Dose-time response prediction

**Fig. 2:**
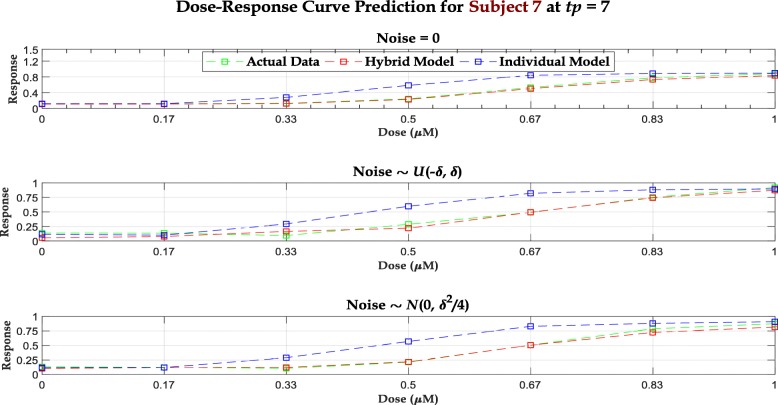
Predicted synthetic data dose-response curves at *t*=7 overlaid with the actual dose-response curves

For the simulation study, we assume that the responses are available for the 7 initial time epochs while we predict for the last epoch with both our proposed hybrid recursive model and a standard RF model for each individual. To train these RFs, we use the protein expressions and responses at *t*<7 as predictors and output respectively and predict for the expression values at *t*=7. We also analyze the effect of noise in response values. Two different scenarios are shown in Fig. 1 along with the noiseless case, where the additive noise values are sampled respectively from $\mathcal {U}\!\left (-\delta, \delta \right)$ & $\mathcal {N}\!\left (0, \textstyle {\frac {\delta ^{2}}{4}} \right)\hspace {-0.3em},\ \delta = 0.05$ and incorporated in (4). Figure 2 displays the predicted dose-response curves overlaid with the actual responses for all 3 scenarios. For objective performance measure, we also perform comparisons between mean square prediction errors (MSPE) for both hybrid and individual models in all 3 scenarios, as illustrated in Table 1.

**Table 1:** Mean square prediction errors (MSPE) for recursive Hybrid Model and Individual RF Models for synthetic data

Subject	Leave-one-out MSPE (×10^−3^)					
	Noiseless		Uniform Noise		Gaussian Noise	
	Hybrid Model	Individual Model	Hybrid Model	Individual Model	Hybrid Model	Individual Model
S1	**0.605**	36.816	**2.778**	38.562	**1.969**	36.952
S2	**1.049**	37.945	**1.529**	36.127	**2.232**	31.976
S3	**1.246**	4.620	**1.538**	5.038	**2.035**	6.337
S4	**0.384**	4.622	**0.505**	3.917	**0.503**	5.418
S5	**1.536**	8.050	**2.889**	9.469	**3.298**	8.597
S6	**1.009**	15.008	**1.119**	18.273	**1.469**	13.589
S7	**0.800**	35.575	**3.153**	37.474	**1.211**	39.244
Mean	**0.947**	20.377	**1.930**	21.266	**1.817**	20.302

Bold values indicate the best performance

### Application on HMS-LINCS data

As mentioned earlier, HMS-LINCS protein expression and mean apoptosis fraction sets contain data for 10 different cell lines at 7 different doses of 5 different drugs. We only keep protein expression data at 24 & 48 hours post drug application in our predictor set and while the response set contains the complete mean apoptosis fraction data. However, only 7 out of 10 cell lines have complete record on apoptosis fractions and these 7 cell lines form our training set. The remaining 3 cell lines with partial records is used to validate our model. Since we are using protein expressions at only 2 times points, we simply put their differences as predictors $\beta _{d, i}^{(p)}$ for the RF regression models (in (17)) to predict *α*_*d*_, *γ*_*d*_.

5 $$ \begin{aligned} {\beta}_{d, i}^{(p)} &= x_{48, d, i}^{(p)} \, - \, x_{24, d, i}^{(p)}, \quad p = 1, 2, \cdots, 14 \\ \hat{\alpha}_{d, i} &= \text{RF}_{\alpha}\left({\beta}_{d, i}^{(1)} \;, {\beta}_{d, i}^{(2)} \;, \cdots, \; {\beta}_{d, i}^{(14)} \right) \\ \hat{\gamma}_{d, i} &= \text{RF}_{\gamma}\left({\beta}_{d, i}^{(1)} \;, {\beta}_{d, i}^{(2)} \;, \cdots, \; {\beta}_{d, i}^{(14)} \right) \end{aligned}  $$

**Fig. 3:**
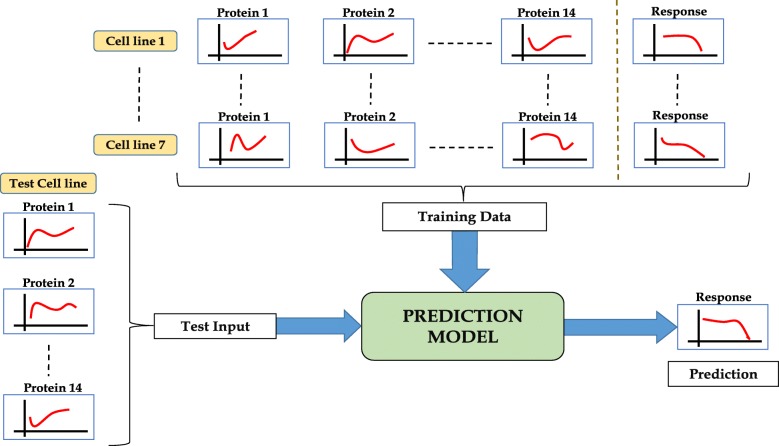
Prediction of dose-response functions of apoptosis fraction from dose-expression functions of multiple proteins

Out of the 3 validation cell lines, 2 cell lines (MMAC-SF and SKMEL28) have apoptosis records for 48 & 72 hours, but the 24 hours records are missing. The other cell line (K2) has apoptosis records for 24 & 48 hours with missing 72 hours records. So, for the former two, we use the 48 hours data as baseline and generate 72 hours prediction while for K2 we use the 24 hours data as baseline and generate 48 hours prediction. A cartoon representation of the prediction procedure is shown in Fig. 3.

**Fig. 4:**
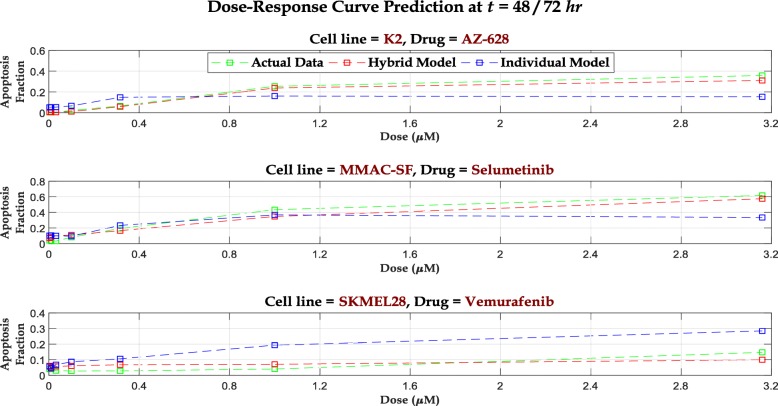
Predicted dose-response curves from Hybrid model and individual RF models at 48/72 hours for 3 representative cell line – drug combinations. The observed dose-response curves are also overlaid

We perform the one-step ahead prediction in (21) for all 5 drugs and calculate the associated mean MSPE over 3 test cell lines, as shown in Table 2. Similar to the simulation study, we compare these results with mean MSPE for standard individual dose-time RF models to put the performance into perspective. For RF training, we use the observed apoptosis fraction at each dose at 72 hours as our responses and the set of protein expressions at the corresponding dose level at 72 hours as our feature set. Since the protein expressions are not available at 72 hours, we fit a *cubic spline* to the expression values at each dose level for the 5 time points available and extrapolate to 72 hours for the required feature set. We use this extrapolated expressions to predict the apoptosis fractions at each dose for cell lines MMAC-SF and SKMEL28. For K2, we use the available 48 hour covariate data to train the RFs and perform prediction. We also plot the predicted dose-response values from both models with the observed values in Fig. 4 for 3 representative cell line – drug combination scenarios.

**Table 2:** Mean square prediction errors (MSPE) for recursive Hybrid Model and Individual RF Models for HMS-LINCS data

Drug	Mean MSPE (×10^−3^)	
	Hybrid Model	Individual Model
AZ-628	**2.790**	19.474
PLX-4720	**6.482**	7.410
SB590885	**2.146**	4.349
Selumetinib	**1.550**	10.556
Vemurafenib	**8.825**	10.538
Mean	**4.359**	10.465

Bold values indicate the best performance

## Discussion

From the MSPE results in Tables 1 and 2, we can infer that the hybrid model predictions fit the actual dose-response curves significantly better than the individual RF models. For synthetic data, the hybrid model shows a mammoth decrease in MSPE values in Table 1 even when noise is present. Specifically, the overall mean MSPE for hybrid model is ∼ 22 times less than the mean MSPE for individual models in noiseless scenario and ∼ 11 times less in noisy cases. For the HMS-LINCS results in Table 2, the overall mean MSPE achieved by our model is ***0.0044***, which is a staggeringly ∼ 2.5 times less than the mean MSPE produced by the individual models (***0.0105***). We can also reach the same conclusion from examining the predicted dose-response curve fits in Figs. 2 and 4. For synthetic data, the predicted curves closely follow the observed dose-response variations even in the presence of significant noise, as demonstrated in Fig. 2 for subject 7 (An additional section illustrates the dose-response predictions for all 7 subjects [see Additional file 1: Figures S4 - S6]). For HMS-LINCS data, we have displayed predictions for 3 representative cases (out of 15 cell line – drug combinations) which also demonstrates the efficacy of our proposed hybrid model. These results bolster our claim that the recursive hybrid model is a much superior predictor of the dose-time response behavior compared to the standard RF methodology.

**Fig. 5:**
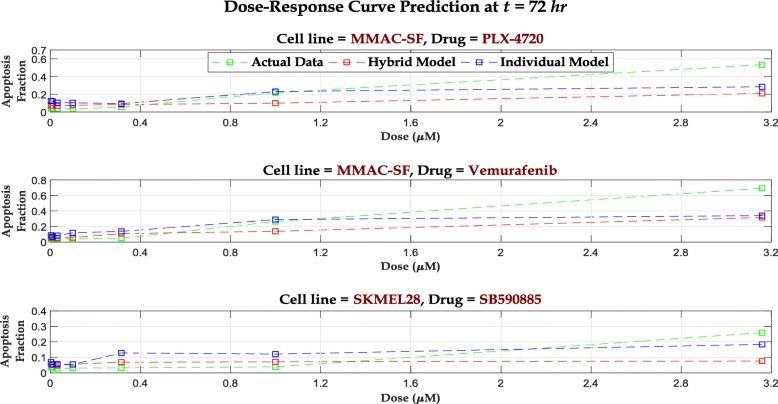
Predicted dose-response curves from Hybrid model and individual RF models at 72 hours for 3 cell line – drug combinations for which RF models outperform the Hybrid model. The observed dose-response curves are also overlaid

One important property of the hybrid model is that– given an initial dose-response curve, all dose-response curves at the subsequent time epochs inherit the theoretical properties of the initial curve (following (13)). Therefore, our model cannot accommodate situations where the properties of dose-response curve changes with *time*. This results in the 3 most glaring mismatches between the observed and predicted dose-response curves for the cell line – drug combinations: *MMAC-SF – PLX4720*, *MMAC-SF – Vemurafenib*, and *SKMEL28 – SB590885*. For these cases, the observed dose-response curves at 72 hours are partially observed sigmoidals where we do not observe the upper asymptotes. Since the Gompertzian kinetics enforces an upper asymptote proportional to the upper asymptote observed at *t*=0 (following (16)), the model becomes ill-scaled. Due to this susceptibility of our model to misspecification, a naïve RF model outperforms our model in all three situations, as shown in Fig. 5 (An additional section shows the predictions for all 15 cases [see Additional file 1: Figures S7 - S9]). Furthermore, although in 12 out of 15 test cases, our hybrid model outperforms RF, there exists some mismatch between the observed and predicted data. The main reason for this discrepancy is the imprecision associated with the estimates of *α*_*d*,*i*_ & *γ*_*d*,*i*_. Observe that for all dose points, we are estimating the two Gompertzian parameters from only 3 time points. With such limited data, the point estimates are bound to have large uncertainties, reflected by the mismatches. However, an increase in time points can reduce the imprecisions as illustrated in simulation study, where we have included 8 time points and observed a much closer fit for the predicted curves from the hybrid model to the observed (synthetic) dose-response curves, even with the presence of noise (Fig. 2).

### Model scalability

The limited nature of HMS-LINCS datasets is precisely the reason we decided on conceptualizing a hybrid model. As mentioned above, to our knowledge, HMS-LINCS is the only publicly available database providing both functional predictor and response data. Several standard ML approaches that have been demonstrated to work well with large pharmacological datasets in literature [10], from where we have chosen Random Forest as our regression model due to its superior predictive capabilities [9, 11] as well as efficient handling of large datasets [14]. Moreover, note that there are only a few functional predictors (*i.e.*, 14) and therefore, in terms of scalability, we could handle a large number of samples with the same number of functional predictors using the standard function-on-function regression easily without much demand on the computational resources. The computational burden may appear to escalate if we increase the number of functional predictors while keeping the sample size fixed, but this will be significantly alleviated by the RF modeling that connects the Gompertzian parameters with the slopes of the predictors. Therefore, the posited model is easily scalable for both *large sample-size* and *large feature-size* without much demand on computational resources. The time aspect is also not problematic since majority of the studies report observations at 72 hours [3, 4]. Given this time horizon, if the temporal resolution is very high, we can always coarsen the resolution at hourly (or daily) scale. The real bottleneck is the number of dose levels which can enforce a large number of optimization operations since the Gompertzian parameters are estimated sequentially at each time point. However, in reality, we do not expect too many dose levels due to the cost involved in data collection at finely resolved dosages.

We, therefore, need to deal with only two effective dimensions of scalability– (a) increase in the number of subjects, and (b) increase in the number of predictors. The model is set up to be simultaneously scalable in both these dimensions. Moreover, the response model for each subject is modeled independently to obtain subject-specific Gompertzian parameters, and therefore, estimation for each subject is trivially parallelizable. Once the parameter estimates are obtained, an RF model is deployed to connect the covariates with response parameters. To put these above discussion into perspective, we can look into the execution time for hybrid model prediction. For our experiments, the synthetic data case roughly takes ∼ 1.1 sec to fit for 6 subjects and predict dose-time responses for subject 7, while for HMS-LINCS data, it takes ∼ 0.785 sec to fit the 7 training cell lines and predict temporal dose-responses for all 3 validation cell lines.

## Conclusions

We have developed a recursive hybrid methodology to model dose-time responses of individuals characterized by a set of functional covariates. Instead of directly connecting the observed responses with the observed covariates, we have taken a *stepwise* approach where the responses are modeled separately according to a parametric specification and the parameters of the response models are connected to the covariates *via* RF regression models. Empirical results suggest that our model provides significant improvement, in terms of MSPE, as compared to a naïve RF model directly connecting responses with covariates. The main strength of our methodology is that it can incorporate additional information about the expected behavior of the responses while letting machine learning methodology drive the processes on which we have no prior information about their expected behavior. We have theoretically shown some desirable properties of our hybrid model and demonstrated its predictive capabilities.

However, all these properties are contingent on proper specification of initial condition. Since all the later dose-response curves inherit the properties of the initial curve, our model cannot account for temporal variation in dose-response curve properties leading to a naïve RF model outperforming it (see Fig. 5). Furthermore, all our predictions are predicated on the availability of a baseline dose-response curve. To handle the situations where an individual comes along with only a set of baseline covariates (say, gene expression), we propose to specify a regression model connecting the baseline dose-response curves in the training set with their respective covariates using either a *functional regression* approach [5] or a fully data-driven *functional random forest* approach [15]. We can then use the covariates of a new individual to predict the associated baseline dose-response curve. This will provide us with the necessary initial conditions to utilize our recursive framework to generate the entire collection of dose-response curves at prespecified time epochs. We propose to investigate this approach in future studies.

We also note that the current model is devised to demonstrate that incorporating some known biological properties can bring about significant improvement in predictions. The model is set up to be computationally fast and easily scalable as number of subjects and predictors increase. However, in order to achieve computational efficiency, we forego the opportunity to perform a full blown likelihood based inference. We are currently investigating a Bayesian hierarchical specification given by

6 $$\begin{array}{*{20}l}  y_{t, d, i} &= \mu_{t, d, i} + \varepsilon_{t, d, i} \end{array} $$

with

7 $$  \begin{aligned} \mu_{t, d, i} &= \mu_{0, d, i} \, e^{\gamma_{d, i} \left(1 \, - \, e^{-\alpha_{d, i} t} \right)} + \eta_{t, d, i} \\ \mu_{0, d, i} &\sim \mathcal{TN} \left(\left[ a_{0} + \frac{b_{0} - a_{0}}{1 + \left(\frac{c_{0}}{d} \right)^{\!\theta_{0}}} \right]\!,\ \sigma_{0}^{2} \right) \end{aligned}  $$

where $\mathcal {TN}(\mu, \sigma ^{2})$ is a *truncated Normal distribution* on an appropriate compact support, while *ε*, *η* are independent Gaussian noises with appropriate supports that match the support of the response. This hierachical model in (6 - 7) offers a formal stochastic extension of the posited hybrid model and the subsequent posterior analysis will allow us to create posterior predictive bands for the test cases, thereby offering insights into the adequacy of our model.

## Methods

The following sections provide a detailed analysis of our proposed Recursive Hybrid Model along with the desired underlying properties.

### Model specification

Let *y*_*t*,*d*,*i*_ be the mean apoptosis fraction at time *t* and dose *d* for subject *i*, *d*=1,2,⋯,*D*; *t*=1,2,⋯,*T*; *i*=1,2,⋯,*m*. We suppress the subscript *i* for the moment and develop a dose-time model for each individual. A simple parametric model for dose-time responses can be written as

8 $$  \begin{aligned} y_{t, d} &= f(t) \, g(d) + \varepsilon \\ \text{where}\\ &f(t) = a_{0} \, e^{\gamma \left(1 \, - \, e^{-\alpha t} \right)} \\ &g(d) = a + \frac{b - a}{1 + \left(\frac{c}{d} \right)^{\!\theta}} \end{aligned}  $$

where *f*(*t*) is a *Gompertz model* in time *t* with *α* controlling the growth rate and *g*(*d*) is the *sigmoidal model* in dose *d* with *a* being the lower asymptotic response at *d*=0, *b* being the upper asymptotic response at *d*=*∞*, *c* is often interpreted as *EC*_50_, *θ* is the Hill slope *i.e.*, the slope at the steepest part of the sigmoidal curve and *ε* is usually assumed to be Gaussian noise. Cross-sectionally, the four parameter sigmoidal function *g*(*d*) is widely used in dose-response studies [16–20] while longitudinally, the two parameter Gompertz model *f*(*t*) is arguably the most popular growth model in tumor modeling efforts [16, 21–24]. Therefore, both *g*(*d*) and *f*(*t*) are well suited if we wish to model the corresponding marginal processes separately. However, assuming a separable product model describing the joint process enforces some unjustifiable assumptions. For instance, such a separable model induces dose invariant temporal growth rate which contradicts Haber’s law [7] and also empirical observations suggesting growth rate at higher dose is significantly different from that observed at small dose levels [6].

To remove separability, we can introduce dose-dependent parameters in *f*(*t*) and time-dependent parameters in *g*(*d*). However, specification of time-dependent parameter makes temporal prediction of responses quite challenging. Furthermore, inclusion of both time-dependent and dose-dependent parameters in Eq. (8) will make the model heavily overparametrized. Therefore, to guarantee the estimability of these parameters, we need to enforce some dependence among these parameters. We capture this dependence by specifying a temporally recursive model for dose-response curves which implicitly induces dependence among the parameters. The recursion relation enables us to generate temporal predictions without incorporating the foregoing dependence explicitly.

### Gompertzian recursion

Broadly speaking, our strategy is to fit a sigmoidal dose-response curve at the first observed time epoch and then enforce the subsequent dose-response curves to follow *Gompertzian law* at each dose point. Gompertzian kinetics essentially suggests exponential growth with exponentially decaying growth coefficient. Several empirical studies have reported Gompertz model to best fit tumor growth data [23, 25, 26]. Now, in order to effectively reduce the size of tumor, the kill rate induced by the drug should mimic the tumor growth pattern and hence we expect the dynamics of apoptosis fraction to follow Gompertzian kinetics too. To illustrate our conceptualization of Gompertzian law, we drop the subscript *d* momentarily. We begin with Ricker’s parametrization [27] of Gompertz model given by

9 $$  n_{t} = n_{0} \, e^{\gamma \left(1 \, - \, e^{-\alpha t} \right)}, \quad \gamma \geq 0, \; \alpha \in \mathbb{R}  $$

where *n*_*t*_ is expected value of the trait (number, density, etc.) under consideration at time *t*, *n*_0_ is the initial value of the trait giving the starting point on the growth curve, *α* is the *growth coefficient* and *γ* controls the upper asymptote of the growth curve (*n*_0_ *e*^*γ*^) by scaling the curve vertically (*i.e.*, the *scaling coefficient*). In our situation, the focal trait is the mean apoptosis fraction. Therefore, our temporal model for the apoptosis fraction is posited as

10 $$  y_{t} = y_{0} \, e^{\gamma \left(1 \, - \, e^{-\alpha t} \right)}, \quad \gamma \geq 0, \; \alpha \in \mathbb{R}  $$

where *y*_0_ is viewed as the mean apoptosis fraction observed immediately after drug application. Now, we can insert the dose subscript *d* in (10) and specify a sigmoidal dose-response model for *y*_0_, *i.e.*

11 $$  y_{0, d} = a_{0} + \frac{b_{0} - a_{0}}{1 + \left(\frac{c_{0}}{d} \right)^{\!\theta_{0}}}  $$

Inserting (11) in (10) yield a model for expected dose-response at time *t* and dose *d* as

12 $$  y_{t, d} = \left[ a_{0} + \frac{b_{0} - a_{0}}{1 + \left(\frac{c_{0}}{d} \right)^{\!\theta_{0}}} \right] e^{\gamma \left(1 \, - \, e^{-\alpha t} \right)} \\  $$

It is easy to see (12) yields the following recursion relation between the apoptosis fractions observed at two consecutive time points at each dose *d*

13 $$\begin{array}{@{}rcl@{}}  y_{t+1, d} = y_{t,d} \, e^{\left[ \gamma \left(1 \, - \, e^{-\alpha} \right) e^{-\alpha t} \right]} \end{array} $$

Now, observe that if we assume *α* and *γ* to be dose invariant in (12), since $e^{\gamma \left (1 \, - \, e^{-\alpha t} \right)}$ is a non-negative quantity, *y*_*t*,*d*_ inherits the sigmoidal property from *y*_0,*d*_. Therefore, the collection of dose-response curves over time are sigmoidal in dose and Gompertzian in time. Furthermore, Eq. (13) connects two sigmoidal curves at consecutive time epochs with the Gompertzian law implicitly inducing temporal dependence among sigmoidal parameters. Evidently, the recursion relation allows prediction in time despite having implicit time-dependent sigmoidal parameters.

### Properties of the recursive model

Observe that in Eq. (12), the assumption of dose-invariant Gompertzian parameters yields an unrealistic scenario. Particularly at *d*=0, the lower asymptotes of the sigmoidal curves shifts with time. It is hard to justify why apoptosis fraction will change with time even though no drug is administered. To alleviate such drawbacks, we make both growth and scaling coefficients dose-dependent. Consequently, (12) becomes

14 $$  \begin{aligned} y_{t, d} &= \left[ a_{0} + \frac{b_{0} - a_{0}}{1 + \left(\frac{c_{0}}{d} \right)^{\!\theta_{0}}} \right] e^{\gamma_{d} \big(1 \, - \, e^{-\alpha_{d} t} \big)} \end{aligned}  $$

while the recursion relation between two consecutive time points at dose *d* is still given by (13) after incorporating *α*_*d*_, *γ*_*d*_. To investigate the asymptotic property of (14), we impose the following reasonable constraints

- Since apoptosis fraction is expected to increase as drug concentration increases, we assume that both *α*_*d*_$\left (\in \mathbb {R}\; \forall d \geq 0 \right)$ and *γ*_*d*_$\left (\in \mathbb {R}^{+}\cup \{0\} \; \forall d\geq 0\right)$ are non-decreasing functions of dose *d*.
- ${{\lim }_{d \, \to \, 0} {\gamma _{d}} = 0}$
- Apoptosis fraction is bounded in [0,1], therefore, we assume that ${{\lim }_{d \, \to \, \infty } {\alpha _{d}} = \alpha ^{*}}$ and ${{\lim }_{d \, \to \, \infty } {\gamma _{d}} = \gamma ^{*}}$ where *α*^∗^, *γ*^∗^ are finite positive constants that satisfy the bounded property of apoptosis fractions.

Under these assumptions, we have the following properties of the dose-response curve at time *t*, obtained from (14).

**Fig. 6:**
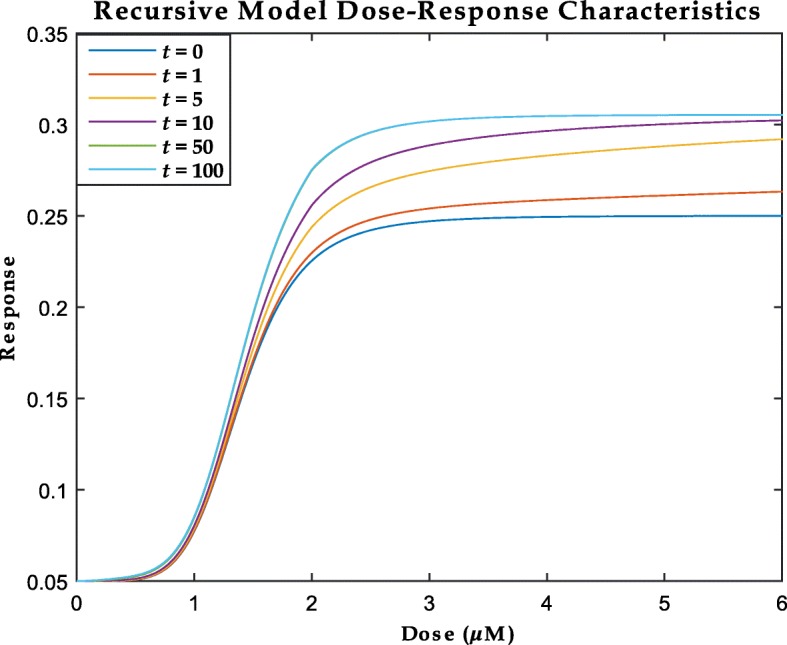
An illustration of the behavior of the recursive Hybrid model developed in Eq. (14) for various dose-time points. The dose-response curve at *t*=50 (*green*) cannot be distinguished from the curve at *t*=100 (*indigo*) demonstrating the asymptotic behavior of the dose-responses curves

(i) At *d*=0, assumption (b) forces the asymptotes of the sigmoidal curves to be stationary at *a*_0_ regardless of time. This is justifiable since we do not expect to see any change in apoptosis fraction from the baseline *a*_0_ without the application of a drug, regardless of how much time has elapsed.
(ii) Given a particular time *t*, assumption (a) makes $\phantom {\dot {i}\!}e^{\gamma _{d} \big (1 \, - \, e^{-\alpha _{d} t} \big) }$ an increasing function of dose *d*. If *y*_0,*d*_ is also an increasing function of *d*, then so is *y*_*t*,*d*_ (following (14)) with lower asymptote at *a*_0_ and upper asymptote at $b_{t} = b_{0} e^{\gamma ^{*} \big (1 \, - \, e^{-\alpha ^{*} t} \big)}$. From assumption (c), *α*^∗^, *γ*^∗^>0, therefore *b*_0_>*a*_0_⇒*b*_*t*_>*a*_0_.
(iii) Since *b*_*t*_ is an increasing function of time, the upper asymptotes of the dose-response curves increases with time indicating the expected greater efficacy at higher dose levels. In general, since the dose-response curves at each time epoch is monotonically increasing in dose (assuming *y*_0,*d*_ is increasing in dose), Eq. (14) suggests dose-response curves at later time points will dominate the curves observed at initial time points. Also, the fact that the apoptosis fraction is bounded above by 1 is easily realized by specifying $b_{0} < e^{-\gamma ^{*}}$.
(iv) Eq. (13) and assumption (c) suggest that
  15 $$\begin{array}{*{20}l}  {\lim}_{t \to \infty} \left[ \frac{y_{t+1, d}}{y_{t, d}} \right] = 1 \end{array} $$
  indicating that the area under the curve (AUC) of the dose response curves do not become arbitrarily large with time but achieves a theoretical steady-state. A graphical representation of the above properties of our proposed recursive model (Eq. (14)) is shown in Fig. 6.

### Regression model for individuals

In the preceding sections, we have discussed the dose-time model for responses observed for a single individual. We have several such individuals, each characterized by a set of protein expressions observed over both dose and time. Let $x_{t, d, i}^{(p)}$ denote the observed expression for protein *p* in subject *i* at dose *d* and time *t*, *p*=1,2,⋯,*P*, *t*=1,2,⋯,*T*, *d*=1,2,⋯,*D*, *i*=1,2,⋯,*m*. We now connect these $x_{t, d, i}^{(p)}$ values with the individual dose-time model specified in (14) by introducing the subscript *i*, which yields

16 $$  \begin{aligned} y_{t, d, i} &= \left[ a_{0, i} + \frac{b_{0, i} - a_{0, i}}{1 + \left(\frac{c_{0, i}}{d} \right)^{\!\theta_{0, i}}} \right] e^{\gamma_{d, i} \left(1 \, - \, e^{-\alpha_{d, i} t} \right)} \\ \end{aligned}  $$

and the recursion relation between consecutive time points for an individual *i* at dose *d* is still given by (13) after incorporating the parameters *α*_*d*,*i*_, *γ*_*d*,*i*_.

For each individual, we begin with fitting a sigmoidal dose-response curve at *t*=0 to estimate *a*_0,*i*_, *b*_0,*i*_, *c*_0,*i*_, *θ*_0,*i*_. We then posit that, for each individual, the growth and scaling coefficients (*α*_*d*,*i*_ & *γ*_*d*,*i*_) are determined by the rate of change in protein expressions over time *i.e.*, fit a temporal trend model for each $x_{\cdot, d, i}^{(p)}$ time series and use the *slope coefficients*$\beta _{d, i}^{(p)} \, \bigg (\,=\,\frac {dx_{\cdot, d, i}^{(p)}}{dt}\!\bigg)$ as the predictors in two Random Forest (RF) regression models in (17). However, since *α*_*d*,*i*_, *γ*_*d*,*i*_ are not observed, we plug their estimates in LHS.

17 $$  \begin{aligned} \alpha_{d, i} &= \text{RF}_{\alpha}\!\left(\beta_{d, i}^{(1)}, \beta_{d, i}^{(2)}, \cdots, \beta_{d, i}^{(P)} \right)\\ \gamma_{d, i} &= \text{RF}_{\gamma}\!\left(\beta_{d, i}^{(1)}, \beta_{d, i}^{(2)}, \cdots, \beta_{d, i}^{(P)} \right) \end{aligned}  $$

The algorithm for the stepwise fitting procedure is given below.

#### Stepwise fitting algorithm

- At *t*=0, fit a sigmoidal curve to the observed dose-responses for each individual *i* and obtain the estimates $\hat {a}_{0, i}$, $\hat {b}_{0, i}$, $\hat {c}_{0, i}$, $\hat {\theta }_{0, i}$ and hence obtain $\hat {y}_{0, d, i}$.
- For each individual in the training set at each dose level, obtain the least square estimates of *α*_*d*,*i*_ & *γ*_*d*,*i*_ using the recursion relation in (13). That is
  18 $$\begin{array}{*{20}l}  {}\left(\tilde{\alpha}_{d, i}, \, \tilde{\gamma}_{d, i} \right) \,=\, \underset{\alpha_{d, i}, \, \gamma_{d, i}}{\text{argmin}} \sum_{t = 1}^{T} \!\left[ y_{t, d, i} \,-\, \hat{y}_{t^{-}, d, i} \, e^{\gamma_{d, i} \left(e^{-\alpha_{d, i} t^{-}} - e^{-\alpha_{d, i} t} \right)} \right]^{2} \end{array} $$
  where $\hat {y}_{t^{-}, d, i}$ is the dose-time response at the immediate preceding time point *t*^−^. Usually, we perform a *one-step ahead prediction* for a specific time epoch *t*, considering *t*^−^=0 and *t*=1 in (18), which yields
  19 $$\begin{array}{*{20}l}  {}\left(\tilde{\alpha}_{d, i}, \, \tilde{\gamma}_{d, i} \right) = \underset{\alpha_{d, i}, \, \gamma_{d, i}}{\text{argmin}} \sum_{t = 1}^{T} \!\left[ y_{t, d, i} \, - \, \hat{y}_{t^{-}, d, i} \, e^{\gamma_{d, i} \big(1 \, - \, e^{-\alpha_{d, i}} \big)} \right]^{2} \end{array} $$
  To satisfy assumption (c), we take the final estimates to be $\hat {\alpha }_{d, i} = \max \!\left (\tilde {\alpha }_{d, i},\ \tilde {\alpha }_{d^{-}, i} \right)$ and $\hat {\gamma }_{d, i} = \max \!\left (\tilde {\gamma }_{d, i},\ \tilde {\gamma }_{d^{-}, i} \right)$ where $(\cdot)_{d^{-}, i}\phantom {\dot {i}\!}$ is the estimate of that parameter obtained at immediately preceding dose level *d*^−^.
- Fit a trend model to the time series of each protein expression for each subject at each dose level and obtain the slope coefficients $\beta _{d, i}^{(1)}, \beta _{d, i}^{(2)}, \cdots, \beta _{d, i}^{(P)}$.
- If the training set consists of *m* individuals, then at each dose *d*, define *m*×1 vectors $\hat {\boldsymbol {\alpha }}_{d},\ \hat {\boldsymbol {\gamma }}_{d}$ and *m*×*P* matrix ***β***_*d*_ as
  20 $$  {}\begin{aligned} \hat{\boldsymbol{\alpha}}_{d} &= \left[\begin{array}{cccc} \hat{\alpha}_{d, 1} & \hat{\alpha}_{d, 2} & \cdots & \hat{\alpha}_{d, m} \end{array}\right]^{T} \\ \hat{\boldsymbol{\gamma}}_{d} &= \left[\begin{array}{cccc} \hat{\gamma}_{d, 1} & \hat{\gamma}_{d, 2} & \cdots & \hat{\gamma}_{d, m} \end{array}\right]^{T} \\ \boldsymbol{\beta}_{d} &= \left[\begin{array}{cccc} \beta_{d, 1}^{(1)} & \beta_{d, 1}^{(2)} & \cdots & \beta_{d, 1}^{(P)} \\ \beta_{d, 2}^{(1)} & \beta_{d, 2}^{(2)} & \cdots & \beta_{d, 2}^{(P)} \\ \vdots & \vdots & \ddots & \vdots \\ \beta_{d, m}^{(1)} & \beta_{d, m}^{(2)} & \cdots & \beta_{d, m}^{(P)} \end{array}\right] = \left[\begin{array}{c} \boldsymbol{\beta}_{d, 1} \\ \boldsymbol{\beta}_{d, 2} \\ \vdots \\ \boldsymbol{\beta}_{d, m} \end{array}\right] \end{aligned}  $$
  Use ***β***_*d*_ as the covariate matrix and $\hat {\boldsymbol {\alpha }}_{d},\ \hat {\boldsymbol {\gamma }}_{d}$ as the output to train the RFs in (17) modeling the Gompertzian parameters.
- To predict for a new individual, use the corresponding matrix $\boldsymbol {\beta }_{d}^{\, \text {new}}$ as input to get $\hat {\boldsymbol {\alpha }}_{d}^{\, \text {new}},\ \hat {\boldsymbol {\gamma }}_{d}^{\, \text {new}}$. Use the observed baseline dose response for this individual and generate temporal forecast using (16). For the one-step ahead prediction in (19), the recursion relation in (13) simplifies as
  21 $$\begin{array}{@{}rcl@{}}  y_{t, d, i} = y_{t^{-}, d, i} \, e^{\, \gamma_{d, i} \big(1 \, - \, e^{-\alpha_{d, i}} \big)} \end{array} $$

## Additional file

Additional file 1 Supplementary information to recursive model for dose-time responses in pharmacological studies. **Figure S1**. Dose-time response surface for synthetic data without noise. **Figure S2**. Dose-time response surface for synthetic data corrupted with Uniform noise. **Figure S3**. Dose-time response surface for synthetic data corrupted with Gaussian noise. **Figure S4**. Predicted dose-response curves at *t*=7 overlaid with the observed dose-response curves for synthetic data without noise. **Figure S5**. Predicted dose-response curves at *t*=7 overlaid with the observed dose-response curves for synthetic data corrupted with Uniform noise. **Figure S6**. Predicted dose-response curves at *t*=7 overlaid with the observed dose-response curves for synthetic data corrupted with Gaussian noise. **Figure S7**. Predicted dose-response curves obtained from Hybrid model and individual RF models at 48 hours for cell line K2. The observed dose-response curves are also overlaid. **Figure S8**. Predicted dose-response curves obtained from Hybrid model and individual RF models at 72 hours for cell line MMAC-SF. The observed dose-response curves are also overlaid. **Figure S9**. Predicted dose-response curves obtained from Hybrid model and individual RF models at 72 hours for cell line SKMEL28. The observed dose-response curves are also overlaid. (PDF 2220 kb)

## Abbreviations

AUC: Area under the curve
HMS-LINCS: Harvard medical school library of integrated network-based cellular signatures
MSPE: Mean squared prediction error
RF: Random forest
