# Supplementary material for: Recursive model for dose-time responses in pharmacological studies
Source: BMC Bioinformatics. 2019 Jun 20;20(Suppl 12):317. doi: 10.1186/s12859-019-2831-4 (PMC6584530; doi:10.1186/s12859-019-2831-4)
Supplement: Supplementary file 1 — Supplementary information to recursive model for dose-time responses in pharmacological studies. Figure S1. Dose-time response surface for synthetic data without noise. Figure S2. Dose-time response surface for synthetic data corrupted with Uniform noise. Figure S3. Dose-time response surface for synthetic data corrupted with Gaussian noise. Figure S4. Predicted dose-response curves at t=7 overlaid with the observed dose-response curves for synthetic data without noise. Figure S5. Predicted dose-response curves at t=7 overlaid with the observed dose-response curves for synthetic data corrupted with Uniform noise. Figure S6. Predicted dose-response curves at t=7 overlaid with the observed dose-response curves for synthetic data corrupted with Gaussian noise. Figure S7. Predicted dose-response curves obtained from Hybrid model and individual RF models at 48 hours for cell line K2. The observed dose-response curves are also overlaid. Figure S8. Predicted dose-response curves obtained from Hybrid model and individual RF models at 72 hours for cell line MMAC-SF. The observed dose-response curves are also overlaid. Figure S9. Predicted dose-response curves obtained from Hybrid model and individual RF models at 72 hours for cell line SKMEL28. The observed dose-response curves are also overlaid. (PDF 2220 kb) [file 12859_2019_2831_MOESM1_ESM.pdf]

RESEARCH

Supplementary information to recursive model for dose-time responses in pharmacological studies

Saugato Rahman Dhruba[1], Aminur Rahman[2], Raziur Rahman[1], Souparno Ghosh[2*] and Ranadip Pal[1]

*Correspondence: souparno.ghosh@ttu.edu
[2]Department of Mathematics and Statistics, Texas Tech University, 1108 Memorial Circle, 79409 Lubbock, TX, USA
Full list of author information is available at the end of the article

Additional figures

In this section, we provide the predicted dose-response curves for the Hybrid model in Eq. (18 - 21) as well as for the individual random forest (RF) models, overlaid with the observed dose-response curves. The two subsections provide figures for both synthetic and HMS-LINCS data, respectively.

Synthetic data fit

Figures S1 – S3 plot the dose-time drug response surfaces in three dimensional space for various levels of additive noise in the response data, while Figures S4 – S6 illustrate the corresponding predicted drug-response curves overlaid with the observed curves.

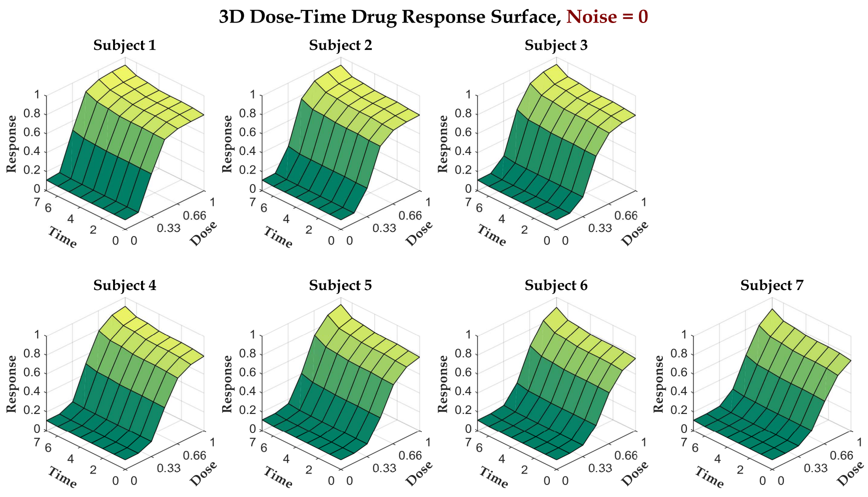

Figure S1: Dose-time response surface for synthetic data without noise.

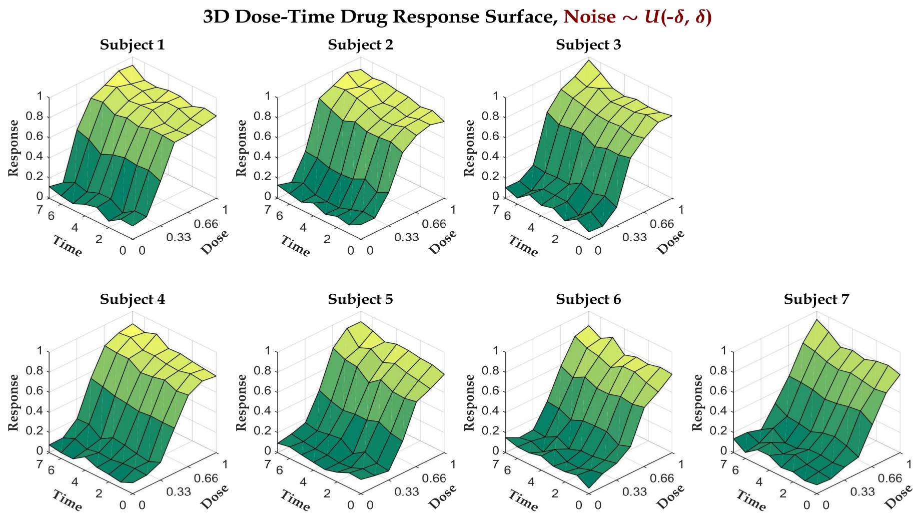

Figure S2: Dose-time response surface for synthetic data corrupted with Uniform noise.

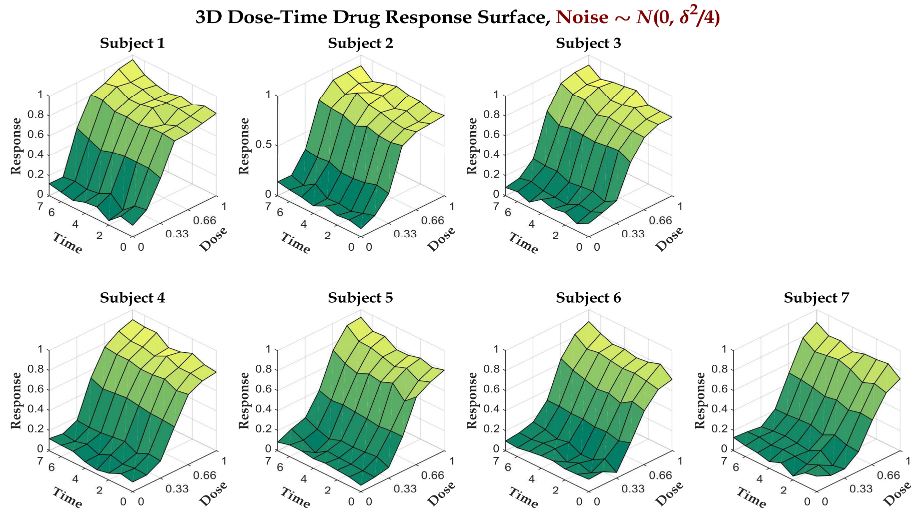

Figure S3: Dose-time response surface for synthetic data corrupted with Gaussian noise.

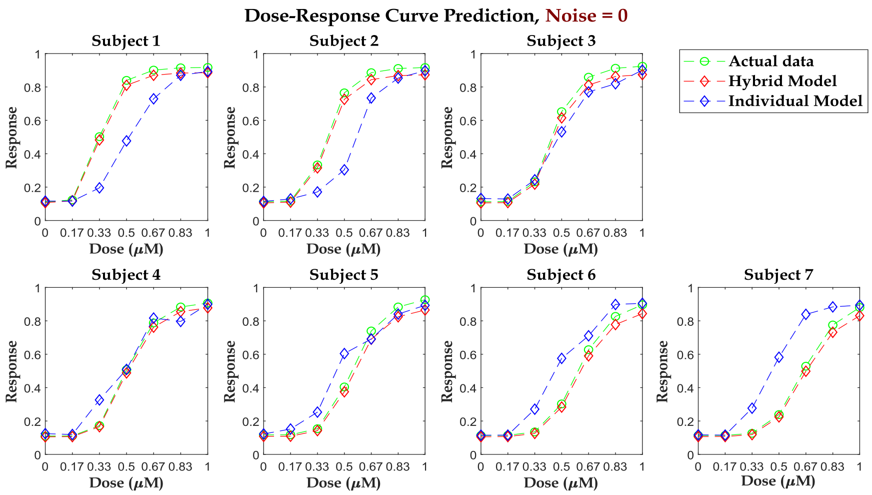

Figure S4: Predicted dose-response curves at $t = 7$ overlaid with the observed dose-response curves for synthetic data without noise.

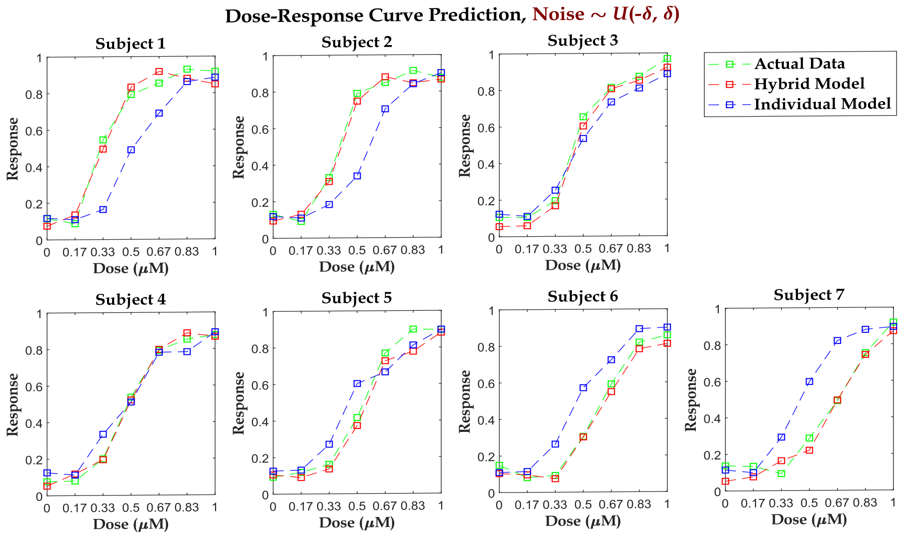

Figure S5: Predicted dose-response curves at $t = 7$ overlaid with the observed dose-response curves for synthetic data corrupted with Uniform noise.

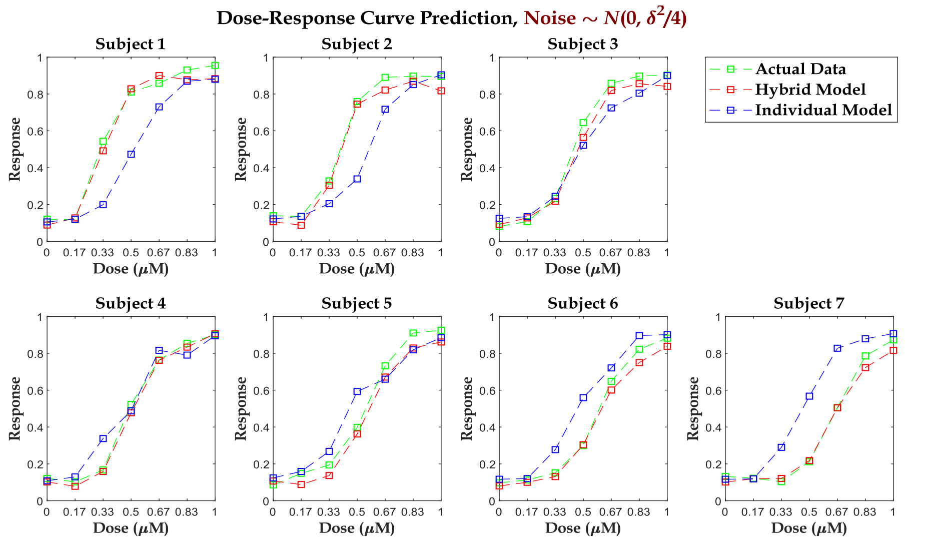

Figure S6: Predicted dose-response curves at $t = 7$ overlaid with the observed dose-response curves for synthetic data corrupted with Gaussian noise.

HMS-LINCS data fit

Figures S7 – S9 shows the predicted drug-response curves overlaid with actual curves for 3 HMS-LINCS validation cell lines – K2, MMAC-SF and SKMEL28.

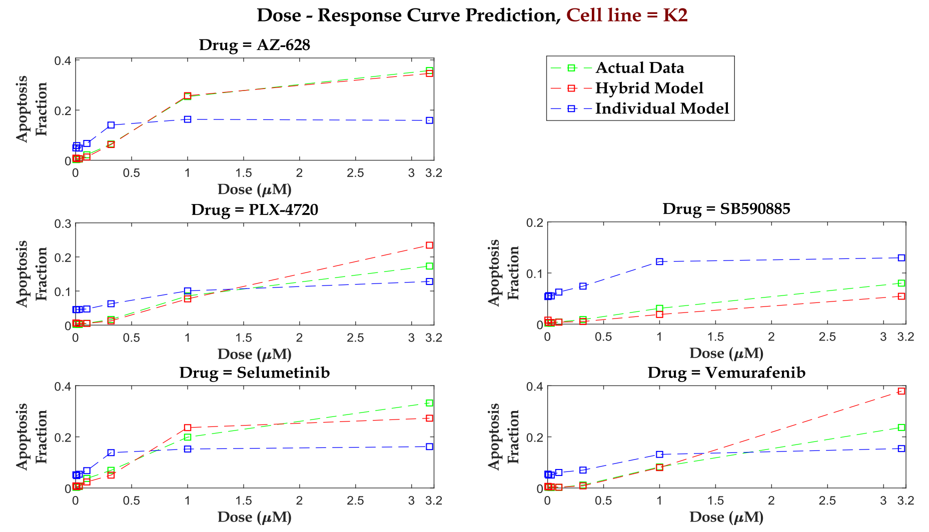

Figure S7: Predicted dose-response curves obtained from Hybrid model and individual RF models at 48 hours for cell line $K2$. The observed dose-response curves are also overlaid.

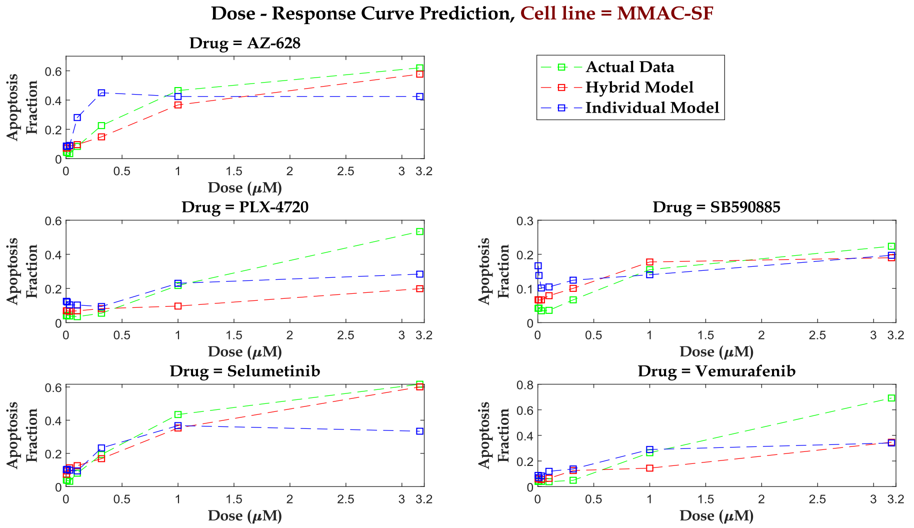

Figure S8: Predicted dose-response curves obtained from Hybrid model and individual RF models at 72 hours for cell line *MMAC-SF*. The observed dose-response curves are also overlaid.

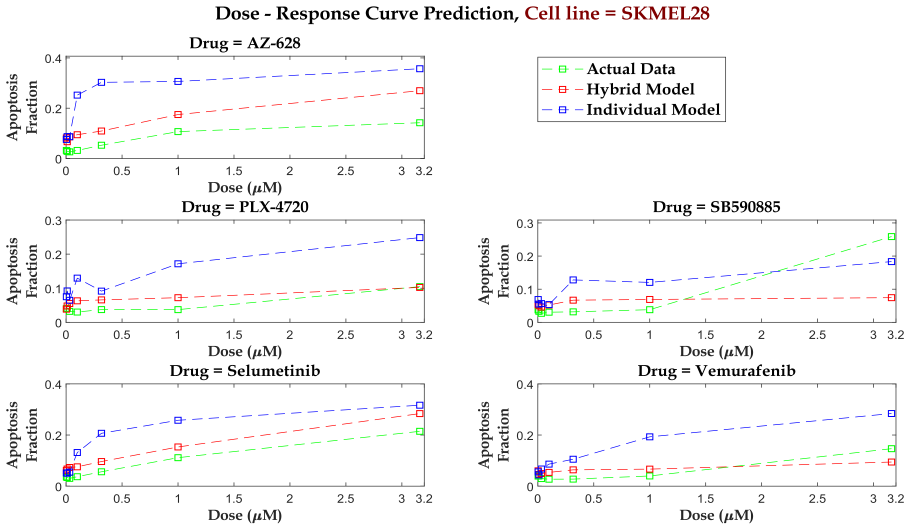

Figure S9: Predicted dose-response curves obtained from Hybrid model and individual RF models at 72 hours for cell line *SKMEL*28. The observed dose-response curves are also overlaid.

Author details
[1]Department of Electrical and Computer Engineering, Texas Tech University, 1012 Boston Ave, 79409 Lubbock, TX, USA. [2]Department of Mathematics and Statistics, Texas Tech University, 1108 Memorial Circle, 79409 Lubbock, TX, USA.
